# Jejunal Metabolic Responses to *Escherichia coli* Infection in Piglets

**DOI:** 10.3389/fmicb.2018.02465

**Published:** 2018-10-16

**Authors:** Hucong Wu, Jiaqi Liu, Siyuan Chen, Yuanyuan Zhao, Sijing Zeng, Peng Bin, Dong Zhang, Zhiyi Tang, Guoqiang Zhu

**Affiliations:** ^1^College of Veterinary Medicine, Jiangsu Co-Innovation Center for Important Animal Infectious Diseases and Zoonoses, Joint International Research Laboratory of Agriculture and Agri-Product Safety of Ministry of Education of China, Yangzhou University, Yangzhou, China; ^2^Guangdong Provincial Key Laboratory of Animal Nutrition Control, Institute of Subtropical Animal Nutrition and Feed, College of Animal Science, South China Agricultural University, Guangzhou, China

**Keywords:** jejunum, metabolism, ETEC, diarrhea, piglet

## Abstract

This study aimed to investigate the jejunal metabolic variations in enterotoxigenic *Escherichia coli* (ETEC)-infected piglets. Piglets were infected with 1 × 10^10^ CFUs (colony-forming units) of ETEC W25K and assigned into diarrheal, recovered, control, and resistant groups. Jejunal samples were harvested at day 6 and metabolic profiles were analyzed via gas chromatography coupled to time-of-flight mass spectrometry (GC/TOFMS). The results showed that 33 metabolites in the jejunum were identified in ETEC-induced diarrhea, including amino acids, fatty acids, sugars, and organic acids. Compared with the control, resistant, and recovered piglets, diarrheal piglets showed higher concentrations of 4-aminobutyric acid (GABA) and glycine in the jejunum. Compared with the control and resistant piglets, six metabolites were markedly decreased in diarrheal piglets, including ornithine, asparagine, glutamine, citric acid, citrulline, and lysine. Collectively, this study provides insights into jejunal metabolic response to ETEC infection and ETEC induced diarrhea in piglets.

## Introduction

Diarrheal illnesses are a severe public health problem and pathogenic enterotoxigenic *Escherichia coli* (ETEC) has been considered as a major cause of diarrhea in human and animals (Fleckenstein et al., 2010). After infection, ETEC rapidly colonizes in small intestine, including duodenum, jejunum, and ileum. ETEC colonization inhibits intestinal immune function and induces inflammatory response. In our previous report, we found that ETEC infection inhibits the mRNA expression of intestinal immune factors, such as polymeric immunoglobulin receptor (pIgR), cryptdin-related sequence 1C (CRS1C), and Reg3γ in mice (Liu et al., 2017). Meanwhile, ETEC infection upregulates intestinal IL-17 and causes dysbiosis of intestinal microbiota via increasing abundance of γ-aminobutyric acid (GABA)-producing *Lactococcus lactis* subsp. *lactis* (Ren et al., 2016b). The jejunal metabolite (e.g., amino acids and polyamine) participate in many important physiological process, such as the regulation of gene expression, synthesis and secretion of hormones, oxidative defense, and so on (Wu, 2009). The proteome analysis from our previous study identifies 92 differentially expressed proteins in the jejunum after exposure to ETEC and large body of these proteins were involved in metabolic process, such as protein turnover, nutrients (i.e., nucleotide, amino acids, carbohydrate, lipid, and inorganic ion) transport and metabolism, coenzyme metabolism, energy production and conversion, and secondary metabolite biosynthesis (Ren et al., 2016a). Metabolomics is an emerging analytical technique to seek global profiles of metabolites in particular samples, including endogenous and exogenous metabolites (Khan et al., 2017). Therefore, we conduct this study to further investigate metabolic profiles in the jejunum after ETEC infection in piglets.

## Materials and Methods

### Bacterial Strains

This study used the *Escherichia coli* F4-producing strain W25K (O149:K91, K88ac; LT, ST, EAST), which was originally isolated from a diarrheal piglet.

### ETEC Infection

This study was conducted according to the guidelines of the Institute of Subtropical Agriculture, Chinese Academy of Sciences. Piglets (Landrace × Yorkshire; 18-day-old) were purchased from ZhengDa, Co., Chongqing, China and orally administrated with ETEC W25K at dose of 1 × 10^10^ CFUs (colony-forming units) for five consecutive days (Ren et al., 2015, 2016a). The control piglets were treated with the same volume of LB medium. Fecal consistency was scored daily as: 0 = normal; 1 = soft; 2 = runny or watery. Piglets with the development of watery diarrhea were defined as diarrheal piglets, and piglets that were recovered from diarrhea were regarded as recovery piglets, while piglets that were challenged with ETEC but not suffered from diarrhea were defined as resistant piglets. Six control piglets, six diarrheal piglets, six recovered piglets, and six resistant piglets were randomly selected for collecting the samples.

### Sample Preparation

Twenty-four piglets were sacrificed at day 6 after ETEC infection and jejunal samples (100 mg) and extraction solvents (50 μL L-2-chlorophenylalanine and 350 μL methanol) were added and then homogenized using a Mini-BeadBeater-16 (Biospec, Co., Bartlesville, OK, United States) for 5 min. The mixture was placed on a shaker at 70°C for 10 min and centrifuged at 12,000 ×*g* and 4°C for 10 min. The supernatant was separated, transferred into a GC vial, and then evaporated to dryness under a stream of N_2_ gas.

Methoxyamine hydrochloride (20 μL, 20 mg/mL pyridine) was added to the dried fraction and incubated at 37°C for 2 h. One hundred μL of bis-(trimethylsilyl) trifluoroacetamide (BSTFA) containing 1% TMCS was rapidly added and incubated at 70°C for 1 h. Then the samples were kept at room temperature before analysis.

### GC-TOFMS Analysis

Metabolites in jejunal samples were derivatized prior to gas chromatography coupled to time-of-flight mass spectrometry (GC-TOFMS) analysis (Agilent 7890A, Agilent, United States; LECO Chroma TOF PEGASUS 4D, MI, LECO, United States). The system utilized a DB-5MS capillary column coated with 5% diphenyl cross-linked with 95% dimethylpolysiloxane (30 m × 250 μm inner diameter, 0.25 μm film thickness; J&W Scientific, Folsom, CA, United States). A 1 μL aliquot of the analyte was injected in splitless mode. Helium was used as the carrier gas, the front inlet purge flow was 3 mL min^-1^, and the gas flow rate through the column was 1 mL min^-1^. The initial temperature was kept at 90°C for 0.25 min, then raised to 240°C at a rate of 5°C min^-1^, and finally to 285°C at a rate of 20°C min^-1^ for 11.5 min. The injection, transfer line, and ion source temperatures were 280, 250 and 220°C, respectively. The energy was -70 eV in electron impact mode. The mass spectrometry data were acquired in full-scan mode with the m/z range of 20–600 at a rate of 100 spectra per second after a solvent delay of 492 s.

### Data Processing and Analysis

Each sample was represented by a GC-TOFMS chromatograph. The GC-TOFMS raw data were processed by Chroma TOF 4.3X software (LECO Corporation, St. Joseph, MI, United States) and LECO-Fiehn Rtx5 database for raw peaks extracting, data baselines filtering and calibration, peak alignment, deconvolution analysis, peak identification, and peak area integration. All the output data exported from Chroma TOF 4.3X software were imported into SIMCA-P software (version 11.0, Umetrics, Umeå, Sweden) for multivariate statistical analyses including a principal component analysis (PCA), partial least squares-discriminant analysis (PLS-DA), and pairwise orthogonal projections to latent structures discriminant analyses (OPLS-DA).

## Results

### PCA Model Analysis

**FIGURE 1:**
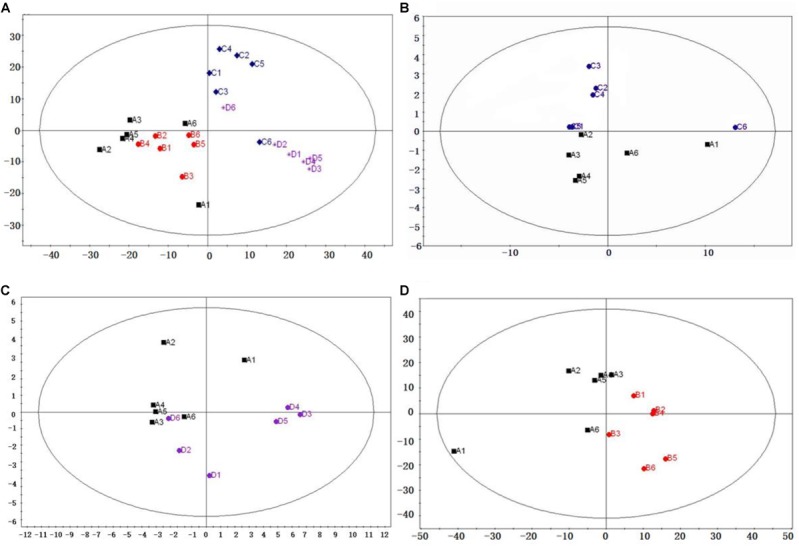
Principal component analysis (PCA) score plot derived from the GC-MS analysis of jejunum from diarrheal piglets (black dots), recovered piglets (red dots), control piglets (blue dots), and resistant piglets (purple dots). **(A)** Total PCA, modeling diagnostic R^2^X = 0.261; Q = 0.0803; **(B)** diarrheal piglets vs. control piglets, modeling diagnostic R^2^X = 0.901; Q = 0.295; **(C)** diarrheal piglets vs. resistant piglets, modeling diagnostic R^2^X = 0.734; Q = 0.379; and **(D)** diarrheal piglets vs. recovered piglets, modeling diagnostic R^2^X = 0.286; Q = –0.163.

Principal component analysis is an unsupervised mathematical procedure used to identify latent structures in the dataset and outliers (Caboni et al., 2014). PCA of jejunal samples from diarrheal piglets, recovered piglets, control piglets, and resistant piglets was shown in **Figure 1A**. The results showed that plots from diarrheal piglets, recovered piglets, control piglets, and resistant piglets were separated each other. As the points that were close to each other had similar metabolic profiles, our results indicated that there might be significant metabolic differences among the four groups. The modeling of the three datasets (diarrheal piglets vs. control piglets, diarrheal piglets vs. resistant piglets, and diarrheal piglets vs. recovered piglets) of separate pairs, revealed separation between subjects (**Figures 1B–D**).

### PLS-DA Model Analysis

**FIGURE 2:**
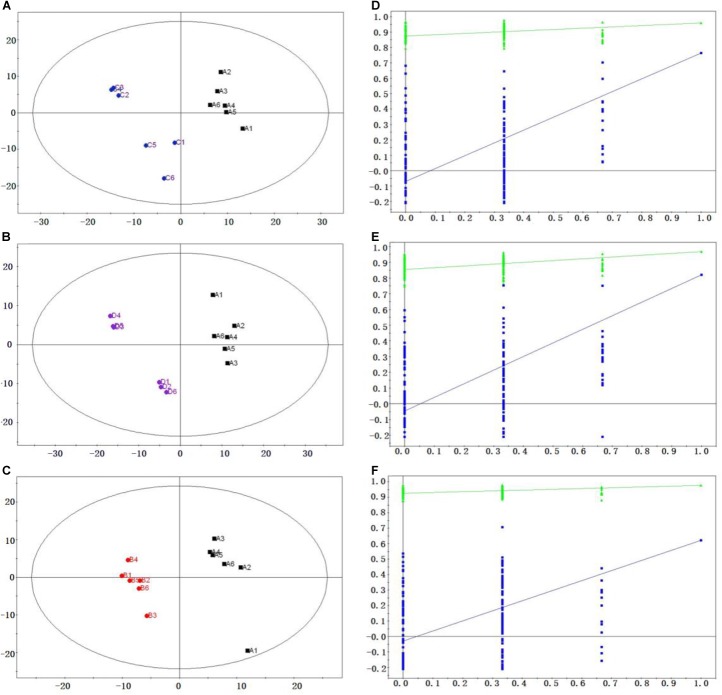
Score plots and permutation test results for PLS-DA model derived from jejunum samples. Score plots of PLS-DA, **(A)** diarrheal piglets (black dots) vs. control piglets (blue dots); **(B)** diarrheal piglets (black dots) vs. resistant piglets (purple dots); **(C)** diarrheal piglets (black dots) vs. recovered piglets (red dots). Permutation validation of PLS-DA model, blue dots and green dots represented Q^2^ and R^2^, respectively; **(D)** diarrheal piglets vs. control piglets; **(E)** diarrheal piglets vs. resistant piglets; and **(F)** diarrheal piglets vs. recovered piglets.

To specify the metabolic variations produced by ETEC infection, PLS-DA models were constructed in jejunal samples (**Figure 2**). The results showed that the samples from each group were perfectly separated in three subjects: diarrheal piglets vs. control piglets (R^2^X = 0.90, Q^2^ = 0.30), diarrheal piglets vs. resistant piglets (R^2^X = 0.73, Q^2^ = 0.38) and diarrheal piglets vs. recovered piglets (R^2^X = 0.29, Q^2^ = -0.16). This phenomenon indicated that the physiological metabolism was interrupted by ETEC infection. In addition, diarrheal piglets showed distinctive metabolic profiles compared with piglets that recovered from diarrhea and were resistant to ETEC infection.

### OPLS-DA Model Analysis

The variable importance in the projection (VIP) statistic of the first principal component of orthogonal partial least squares discriminant analysis (OPLS-DA) model (threshold > 1) coupled with the *P*-value of the Student’s *t*-test (threshold < 0.05) were used for selecting significant variables responsible for group separation.

**FIGURE 3:**
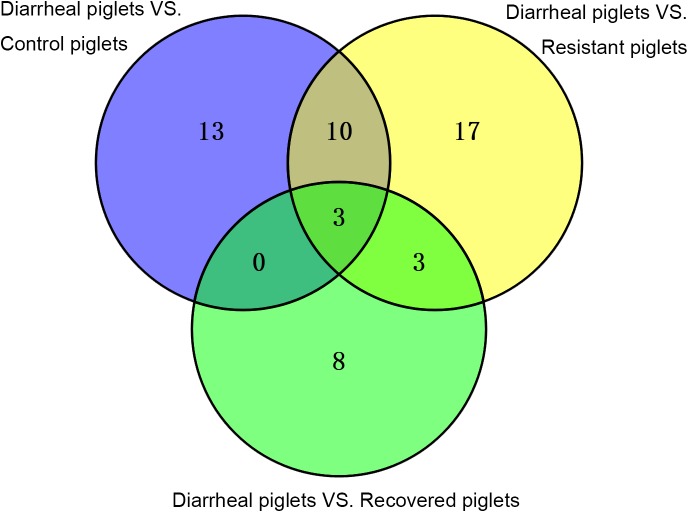
Venn diagrams illustrating that similar differential metabolites (including increase and decrease) among diarrheal piglets vs. control piglets, diarrheal piglets vs. recovered piglets, and diarrheal piglets vs. resistant piglets.

**FIGURE 4:**
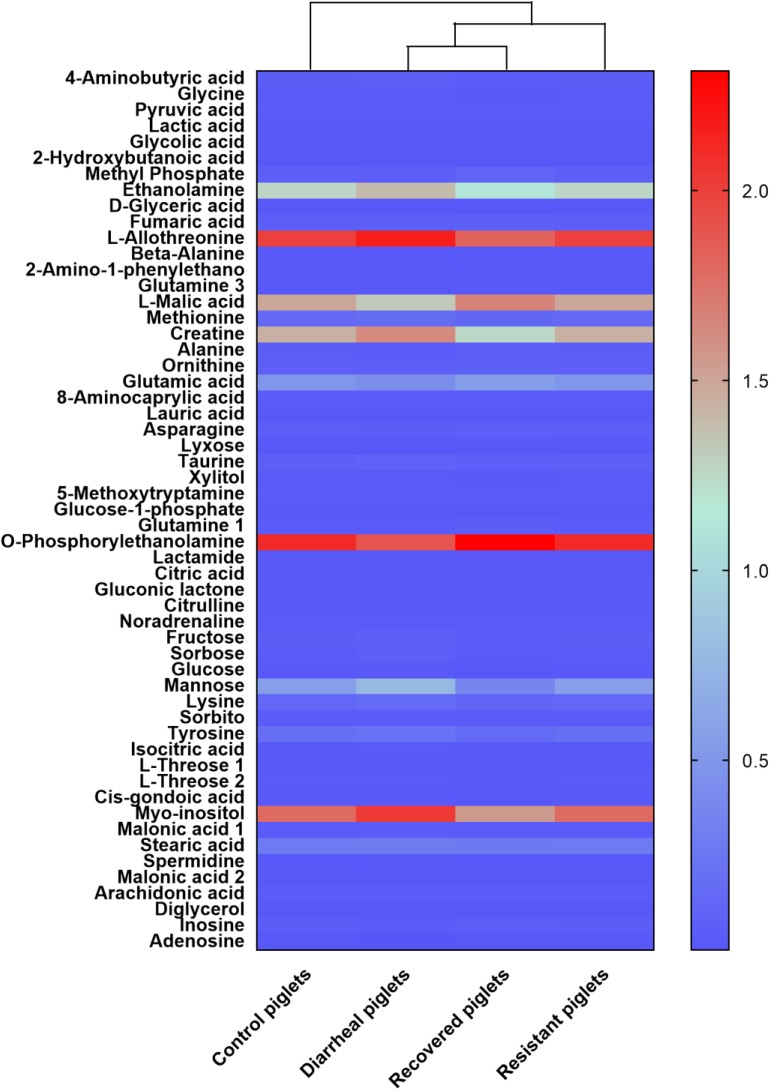
The results from the analysis of differential metabolites were normalized by logarithmic and Euclidean distance and produced a heat map.

As shown in **Figures 3**, **4**, the OPLS-DA models showed a clear separation between the diarrheal piglets vs. control piglets (R^2^X = 0.41, R^2^Y = 0.96, Q^2^ = 0.77), diarrheal piglets vs. resistant piglets (R^2^X = 0.45, R^2^Y = 0.97, Q^2^ = 0.82) and diarrheal piglets vs. recovered (R^2^X = 0.32, R^2^Y = 0.98, Q^2^ = 0.62). We detected 26, 33, and 14 differential metabolites between diarrheal piglets vs. control piglets, diarrheal piglets vs. resistant piglets and diarrheal piglets vs. recovered piglets, respectively. However, only three differential metabolites were commonly altered (including increase and decrease) among them.

Compared with the control piglets, diarrheal piglets showed higher concentration of nine metabolites in the jejunum [4-aminobutyric acid (GABA), glycine, 8-aminocaprylic acid, taurine, 5-methoxytryptamine, lactamide, isocitric acid, L-threose, and malonic acid]. However, 17 metabolites showed a decreased trend in the diarrheal piglets (2-hydroxybutanoic acid, L-allothreonine, 2-amino-1-phenylethanol, methionine, ornithine, lauric acid, asparagine, glutamine, *O*-phosphorylethanolamine, citric acid, citrulline, lysine, tyrosine, myo-inositol, stearic acid, spermidine and arachidonic acid) (**Table 1**).

**Table 1:** The variation in content of metabolites in the jejunum between diarrheal and control piglets.

Metabolites	RT	Cout	Mass	VIP	*P*-value	Fold change
Lactamide	16.08	12	257	1.81	0.002	271000.00
4-Aminobutyric acid^∗^	5.53	17	69	1.92	0.001	4.33
8-Aminocaprylic acid^∗^	13.60	24	174	1.89	0.001	3.10
5-Methoxytryptamine	15.27	24	174	1.42	0.032	2.71
L-Threose 2	19.48	24	73	1.44	0.029	2.28
Taurine	14.20	24	326	1.58	0.013	2.25
Isocitric acid	18.71	24	147	1.61	0.011	1.99
Glycine	7.02	24	102	1.62	0.011	1.98
Malonic acid 1	20.77	23	122	1.42	0.031	1.67
2-Amino-1-phenylethanol	11.19	24	156	1.49	0.022	0.84
L-Allothreonine	10.18	24	73	1.49	0.023	0.80
Methionine	12.04	24	176	1.41	0.034	0.77
Tyrosine	18.37	24	218	1.37	0.040	0.71
Ornithine	13.31	24	142	1.52	0.018	0.68
Myo-inositol	20.78	24	217	1.54	0.016	0.66
Citrulline	16.51	24	157	1.70	0.006	0.64
Lysine	18.09	24	156	1.51	0.019	0.64
Asparagine^∗^	14.12	24	116	1.93	0.001	0.56
Stearic acid	22.74	24	117	1.56	0.015	0.54
Citric acid	16.37	24	273	1.38	0.038	0.53
*O*-Phosphorylethanolamine	15.87	24	73	1.67	0.008	0.51
2-Hydroxybutanoic acid	7.06	19	146	1.38	0.039	0.50
Arachidonic acid	25.75	24	80	1.83	0.003	0.38
Glutamine 1	15.74	24	156	1.55	0.016	0.31
Lauric acid	13.94	16	117	1.55	0.016	0.27
Spermidine	22.83	20	174	1.41	0.033	0.25

*^∗^The extremely significant metabolites between diarrheal and control piglets, which *P*-value < 0.001*.

Compared with the resistant piglets, nine metabolites were significantly enhanced in the diarrheal piglets (GABA, glycine, pyruvic acid, lactic acid, ethanolamine, creatine, 8-aminocaprylic acid, taurine, and noradrenaline), while 24 metabolites were significantly enhanced in the diarrheal piglets (beta-alanine, glutamine, L-malic acid, alanine, ornithine, glutamic acid, asparagine, lyxose, glucose-1-phosphate, citric acid, gluconic lactone, citrulline, fructose, sorbose, mannose, lysine, sorbitol, L-threose, spermidine, malonic acid, diglycerol, inosine, uridine monophosphate, and lactobionic acid) (**Table 2**).

**Table 2:** The variation in content of metabolites in the jejunum between diarrheal and resistant piglets.

Metabolites	RT	Count	Mass	VIP	*P*-value	Fold change
Lactic acid	6.38	10	174	1.43	0.011	1498647.03
4-Aminobutyric acid^∗^	5.53	17	69	1.88	0.000	3.99
Glycine^∗^	6.06	24	102	1.88	0.000	3.89
Taurine	14.20	24	326	1.57	0.003	3.25
8-Aminocaprylic acid	13.60	24	174	1.30	0.026	2.10
Creatine	12.58	24	115	1.56	0.004	1.86
Ethanolamine	8.81	24	174	1.38	0.015	1.84
Noradrenaline	17.05	24	174	1.55	0.004	1.75
Pyruvic acid	6.20	24	59	1.34	0.020	1.44
Glutamine 3	11.39	22	155	1.40	0.013	0.76
L-Malic acid	11.49	24	73	1.28	0.028	0.73
Glutamic acid	13.39	24	246	1.50	0.006	0.72
beta-Alanine	10.81	24	248	1.26	0.031	0.70
Lysine	18.09	24	156	1.30	0.025	0.65
Asparagine^∗^	14.12	24	116	1.65	0.001	0.64
Ornithine	13.32	24	142	1.32	0.022	0.63
Citrulline	16.52	24	157	1.42	0.011	0.60
Alanine	13.22	22	188	1.33	0.021	0.53
L-Threose 1	19.24	24	147	1.29	0.027	0.51
Glucose-1-phosphate^∗^	15.57	24	217	1.81	0.000	0.48
Inosine	25.13	24	217	1.50	0.006	0.47
Gluconic lactone	16.51	22	56	1.22	0.039	0.41
Sorbitol	18.25	24	147	1.40	0.014	0.31
Spermidine	22.83	20	174	1.46	0.008	0.30
Sorbose	17.33	24	103	1.31	0.023	0.25
Lyxose	14.14	17	103	1.32	0.023	0.25
Fructose	17.18	24	103	1.32	0.023	0.25
Citric acid	16.37	24	273	1.61	0.002	0.22
Mannose	17.94	24	73	1.24	0.035	0.20
Diglycerol	24.42	11	103	1.20	0.044	0.15
Malonic acid 2	23.50	16	47	1.52	0.005	0.13
Lactobionic acid	27.80	14	73	1.24	0.034	0.12
Uridine monophosphate	27.36	16	169	1.48	0.007	0.11

*^∗^The extremely significant metabolites between diarrheal and resistant piglets, which *P*-value < 0.001*.

Compared with the recovered piglets, 14 metabolites were significantly different in diarrheal piglets, and 8 metabolites were increased in the jejunum (GABA, glycine, glycolic acid, D-glyceric acid, xylitol, glucose, *cis*-gondoic acid, and malonic acid). Meanwhile, six metabolites were decreased in diarrheal piglets (methyl phosphate, fumaric acid, alanine, inosine, adenosine, and uridine monophosphate) (**Table 3**).

**Table 3:** The variation in content of metabolites in the jejunum between diarrheal and recovered piglets.

Metabolites	RT	Count	Mass	VIP	*P*-value	Fold change
Glucose	17.42	24	205	1.79	0.016	2.20
D-Glyceric acid	9.47	24	189	1.85	0.012	2.11
Glycine	6.06	24	102	2.04	0.004	1.85
4-Aminobutyric acid	5.53	17	69	1.91	0.009	1.75
Xylitol	14.58	24	103	1.69	0.027	1.61
*cis*-Gondoic acid	19.80	24	117	1.64	0.032	1.58
Malonic acid	20.78	23	122	1.60	0.038	1.38
Glycolic acid	6.49	24	177	1.65	0.031	1.30
Fumaric acid	9.76	24	245	1.67	0.028	0.74
Methyl phosphate	7.66	24	241	1.77	0.018	0.59
Inosine	25.13	24	217	1.96	0.006	0.52
Alanine	13.22	22	188	1.64	0.033	0.42
Adenosine	25.59	21	230	1.70	0.026	0.26
Uridine monophosphate	27.36	16	169	2.30	0.000	0.09

## Discussion

Infection with ETEC bacteria is the major cause of diarrhea in human and animals. After infection, ETEC rapidly colonizes the intestine and secretes exotoxins, which further disrupt intestinal barrier integrity and cause secretory diarrhea (Deng et al., 2015). In addition, ETEC colonization induces imbalance of intestinal microbiota and may dysregulate intestinal metabolism (Ren et al., 2016b). In this study, 33 metabolites have been identified in ETEC induced diarrhea, including amino acids, fatty acids, sugars, and organic acids.

Compared with the control, resistant and recovered piglets, diarrheal piglets have higher concentrations of GABA and glycine in the jejunum. GABA, a transmitter of enteric interneurons, has been noticed in the cytoplasm and the brush border of intestinal epithelial cells and regulates the function of the gastrointestinal tract (Wang et al., 2004; Li et al., 2012; Jung et al., 2017). The direct functions of intestinal GABAergic signaling system have been identified to be involved in fluid transport through luminal secretion of Cl^-^ (Jin et al., 2006), which is a major driving force for fluid secretion and increased during diarrhea. Similar to our results in piglets, Li et al. (2012) reported that intestinal GABAergic signaling was upregulated in diarrheal mice caused by ovalbumin and blocking this GABA signaling decreased the occurrence of allergic diarrhea. In our previous study, we found that ETEC infection increased GABA-producing *L. lactis* subsp. *lactis* and GABA production, which further promotes IL-17 expression through mTORC1–S6K1–EGR-2–GFI-1 pathway and mediates intestinal inflammation (Ren et al., 2016b). Glycine serves as a precursor for glutathione along with cysteine and glutamic acid (Yin et al., 2016), while cysteine, glutamic acid and glutamine were markedly decreased in diarrheal piglets, suggesting that increased glycine failed to contribute to the glutathione synthesis and antioxidant effect in ETEC model. In intestinal injury, even after manifestation of a severe systemic impairment (Effenberger-Neidnicht et al., 2014) and causing pH-dependent membrane damage to ETEC (Vanhauteghem et al., 2012), glycine has been demonstrated to protect intestine against subacute endotoxemia. Meanwhile, the glycine cleavage system contributes to the intracellular replication of virulent bacterium and pathogenesis (Brown et al., 2014). Thus, the increased GABA and glycine in the jejunum may mediate or promote diarrhea in ETEC infectious piglet model.

Compared with the control and resistant piglets, we found that six metabolites were markedly decreased in diarrheal piglets, including ornithine, asparagine, glutamine, citric acid, citrulline, and lysine. Ornithine, asparagine, and citrulline play roles in the urea cycle (Mikulski et al., 2015), thus decreased ornithine and asparagine may indicate urea cycle is altered in ETEC induced diarrhea. Aspartate, a precursor of asparagine, has been demonstrated to enhance intestinal integrity and energy status in weaning piglets after lipopolysaccharide challenge (Pi et al., 2014) and alleviate diquat-induced intestinal oxidative stress (Yin et al., 2015). Thus, the decreased jejunal asparagine in diarrheal piglets may exacerbate ETEC infection. Glutamine has been indicated to modulate intestinal permeability and tight junction expression in various diseases, and serves as a protective mechanism against radiation-induced diarrhea and diarrhea-predominant irritable bowel syndrome (Kucuktulu et al., 2013; Bertrand et al., 2015). Lower jejunal glutamine in diarrheal piglets suggested that ETEC infection influenced glutamine synthesis, which may further disturbs the protective mechanism of glutamine against diarrhea. Citrate is an intermediate in the tricarboxylic acid cycle (TCA) and the reduced citrate in the diarrheal piglets suggested that the TCA was altered after ETEC infection.

Out of our expectation, we found that the lactic acid in diarrheal piglets was much higher than resistant piglets as a 1498647 fold change. Lactic acid was the metabolite fermented by various intestinal microorganisms (e.g., *Klebsiella, Bacteroides, Lactobacillus*), and presented in the intestine lumen (Zhao et al., 2011). As mentioned above, ETEC infection increased the relative abundance of lactic acid-producing bacteria (*L. lactis* subsp. *lactis*) significantly, thus the concentration of lactic acid in jejunum lumen was increased. But it failed to explain why lactic acid in jejunum tissue of diarrheal piglets was much higher than resistant piglets. In the healthy condition, the intestine of mammals was incapable of absorbing lactic acid, while the intestinal permeability might be changed in some pathological conditions, and the lactic acid was permitted to permeate the intestinal mucosa, thus the lactic acid may be regarded as an important indictor response to the intestinal barrier function (Qiu et al., 2013; Ikuta et al., 2017). We concluded that ETEC infection might impair the intestinal barrier of piglets and increase the permeability of jejunum (Yang et al., 2014), thus resulted in the high concentration of lactic acid in jejunum tissue.

## Ethics Statement

All experimental protocols were approved by the Animal Care and Use Committee of Yangzhou University [approval ID: SYXK (Su) 2005-0005]. All animal care and use protocols in this study were performed in accordance with the approved current guidelines. At the end of the experimental period, all of the surviving or sick piglets were euthanized by potassium chloride.

## Author Contributions

GZ conceived and designed the study. HW, JL, SC, YZ, SZ, PB, and DZ conducted experiments. HW and JL carried out animal experiments, performed statistical analysis, and drafted the manuscript. GZ finalized the manuscript. ZT conceived and designed the study and finalized the manuscript. All authors read and approved the final version of the manuscript.

## Conflict of Interest Statement

The authors declare that the research was conducted in the absence of any commercial or financial relationships that could be construed as a potential conflict of interest.
